# Power Loss Characteristics of a Sensing Element Based on a Polymer Optical Fiber under Cyclic Tensile Elongation

**DOI:** 10.3390/s110908741

**Published:** 2011-09-08

**Authors:** Yung-Chuan Chen, Li-Wen Chen, Wei-Hua Lu

**Affiliations:** 1 Department of Vehicle Engineering, National Pingtung University of Science and Technology, Pingtung 91201, Taiwan; E-Mails: chuan@mail.npust.edu.tw (Y.-C.C.); liwen@mail.npust.edu.tw (L.-W.C.); 2 Department of Material Engineering, National Pingtung University of Science and Technology, Pingtung 91201, Taiwan

**Keywords:** plastic optical fiber, cyclic tensile loading, displacement sensor

## Abstract

In this study, power losses in polymer optical fiber (POF) subjected to cyclic tensile loadings are studied experimentally. The parameters discussed are the cyclic load level and the number of cycles. The results indicate that the power loss in POF specimens increases with increasing load level or number of cycles. The power loss can reach as high as 18.3% after 100 cyclic loadings. Based on the experimental results, a linear equation is proposed to estimate the relationship between the power loss and the number of cycles. The difference between the estimated results and the experimental results is found to be less than 3%.

## 1. Introduction

Compared with conventional glass fibers, polymer optical fiber (POF) offers a larger core diameter, higher numerical aperture, greater flexibility, lighter weight, easy coupling and lower cost [1,2]. In recent years, the use of optical fiber sensors for measuring displacement, dynamic response, and other possible applications has become increasingly common [3–6]. Babchenko and Maryles [3] developed a displacement sensor with a bent imperfect POF and demonstrated that its sensitivity was significantly enhanced by the presence of the imperfection. Kuang *et al.* [4,5] proposed ways for monitoring vertical deflection in concrete beams and monitoring dynamic response in composite beams using POF sensors in which a segment of the cross-sectional profile was deliberately removed in order to enhance the sensing performance. Lomer *et al.* [6] presented a quasi-distributed level sensor based on an extra attenuation caused by bending and polishing plastic optical fibers. The level sensor takes advantage of the imperfection and the different revolving spindle to increase the sensitivity. Kulkarni *et al*. [7] investigated the use of a novel POF sensor for measuring force in terms of weight. It was shown that increasing the corrugation pitch of the deforming plates enhanced microbend sensitivity. Kuang *et al.* [8] developed a high sensitivity and easy fabricated plastic optical fiber (POF) displacement sensor based on dual cyclic bending.

In recent years, many researchers have investigated the power attenuation (*i.e.*, sensitivity) characteristics of POF sensors [9–17]. Arrue *et al.* [9] studied the effect of the bend radius on the power loss induced in POFs. The results indicated that the power loss increased as the bend radius was reduced. Losada *et al*. [10–12] showed that applying strain to a multiple-curvatured POF could increase its power loss. Daum *et al.* [13] presented an experimental investigation into a tensile loading of POF and showed that the power loss was affected by only 2% to 3% prior to the occurrence of plastic deformation of the fiber. In their previous studies [14–17], the authors have performed experimental and numerical investigations into the power attenuation characteristics of bent and elongated POFs, and found that the power loss increased significantly as bend radii was reduced or the elongation increased. Based on these results, the authors suggested that for fabricating a POF-based sensor the sensitivity of the device can be enhanced by applying an appropriate elongation to the POF specimen.

Up to now, little information regarding the sensitivity of POFs subjected to cyclic tensile loading has been revealed in the published literature. The aim of this study was thus to discover the power attenuation characteristics of POF fibers subjected to cyclic tensile loading. A series of experimental tests were performed to evaluate the power losses induced in cyclic elongated POFs with various load levels. One of the possible applications of this research may be development of a sensor which is buried in a structure to measure a small displacement at certain spots or in small regions, e.g., the contact points between a bridge and its piers.

## 2. Burgers Model

**Figure 1.**
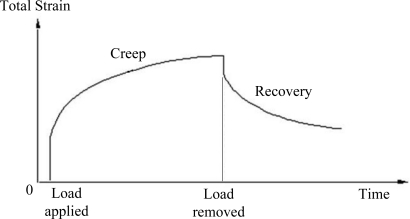
The strain-time relationship of Burgers’ model.

The polymer material is viscoelastic. For the most general case of a linear viscoelastic material, the total creep strain ɛ(t) is given by Burgers’ model, which is a combination of the Maxwell and Kelvin models [18]:

(1) $$ɛ(t)={ɛ}_{1}+{ɛ}_{2}+{ɛ}_{3}=\frac{\sigma }{E_{1}}+\frac{\sigma }{E_{2}}\left(1-\text{exp}\left(\frac{-E_{2}t}{\eta_{2}}\right)\right)+\frac{\sigma t}{\eta_{3}}$$

where ɛ_1_ is the instantaneous elastic strain, ɛ_2_ is the delayed elastic strain [18] and ɛ_3_ is the Newtonian flow viscoelastic strain, which is identical to the strain of a viscous liquid following Newton’s law of viscosity. E_1_ and E_2_ are the elastic moduli, η_2_ and η_3_ are viscosities, σ is the applied stress, and t is the creep time. The strain-time relationship of Burgers’ model [19] is shown in Figure 1.

## 3. Experimental Setup

**Figure 2.**
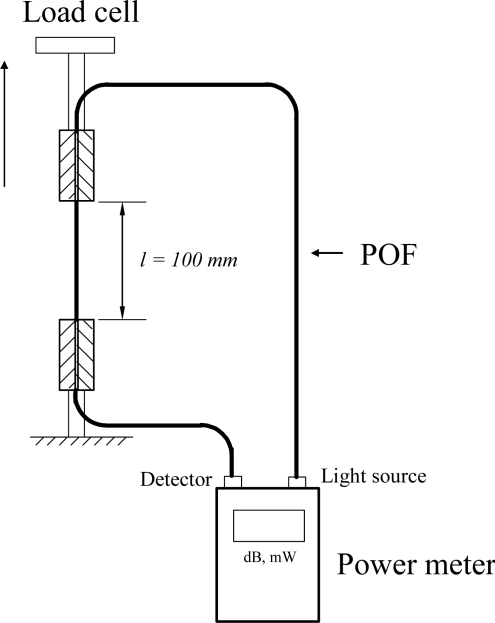
Experimental setup used to measure power loss in cyclic tensile test POF specimen.

Figure 2 presents a schematic illustration of the experimental setup used to measure the power losses in the polymer optical fibers under cyclic tensile loading. As shown, the setup includes a tensile test machine (EZ Test-500N, Shimadzu, Kyoto, Japan), a computer system and an optical power meter (Photom, model 205A, Tokyo, Japan).

The POF specimen is a step index type SH-4001 fiber (Mitsubishi Rayon Company Ltd.) with a coating diameter of 2.2 mm, a cladding diameter of 1 mm, a core diameter of 0.98 mm, and a numerical aperture (NA) of 0.5. The refractive index of the core and cladding are 1.492 and 1.402, respectively. The core, cladding and coating of these POFs are fabricated from polymethyl methacrylate (PMMA), fluorinated polymer and low-density polyethylene (LDPE), respectively. In experiments, a length of 700 mm of POF is cut off from a scroll. The middle of the POF line is carefully measured to have a length of exact 100 mm and then clamped vertically on the test kit as the test section. Two ends of the POF line are connected to the optical power meter. One is connected to the light source (a light emitting diode with a wavelength of 660 nm), and the other is connected to the power detector. The launch NA of the LED used is 0.5. The power delivered to a POF specimen before elongating is measured in advance and denoted as P_in_. The output power measured during fiber being elongated is denoted as P_out_. The test result shows that the power P_in_ from the LED to the optical fiber is about 65 μW and this value is used for power normalization. In this study, the power losses in the POF specimens subjected to cyclic tensile loading are explored. In accordance with the JIS C6861 standard for plastic optical fibers test, the elongation rate is V = 100 mm/min in experiments. Here, one cycle of tensile loading is defined as the test section of the specimen is elongated with a load level varies from 0 N to P N at an elongation rate of V, and then is released to 0 N at the reverse rate of elongation. The cyclic tensile tests are performed with four different load levels, *i.e.*, P = 70, 80, 90 and 100 N, at the temperature of T = 25 °C. In each experiment, the number of cycles (C_y_) is 100.

**Figure 3.**
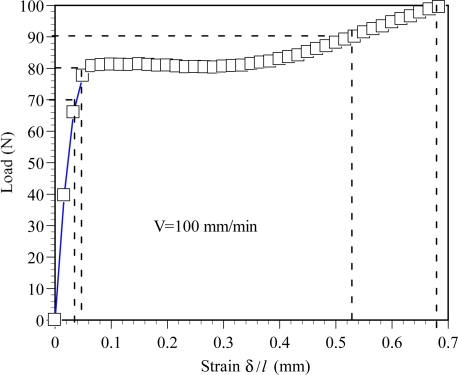
The load-elongation curve of the POF specimen.

Figure 3 shows the load-displacement curve of the POF specimen. It can be seen that the yielding load of the used POF specimen is about 81 N. Therefore, in this study, four load levels, *i.e.*, P = 70, 80, 90 and 100 N, are used to investigate the effect of load level on the power loss in POF specimens. Two of them, *i.e.*, P = 90 and 100 N, exceeded the 81 N yielding load. It can be understood from the experimental tests how larger loads can affect power losses.

**Figure 4.**
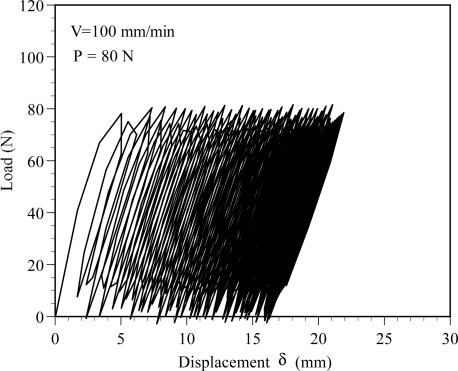
The cyclic load-displacement curve at load level P = 80 N.

Figure 4 presents the experimental cyclic tensile load-elongation results at a load level of P = 80 N. Due to the data sampling frequency of 1 Hz, some details of the experimental data may not be recorded. Thus, in Figure 4, some of cyclic results do not show the load of 80 N or 0 N. During the cyclic tensile process, the resulting power losses in the POFs are detected continually using the power meter and processed using a PC.

## 4. Results and Discussions

According to Burgers’ model, it can be seen from Figure 1 that the retarded elastic deformation and permanent deformation are still retained in the POF specimen when the applied loading is removed. The recovery of retarded elastic deformation begins when the load is released and it takes a period of time to finish the process. This is because the retarded elastic deformation arises from the transformation of a given equilibrium conformation into an elongated structure. The applied load level shown in Figure 4 is 80 N, which is lower than the yielding load of the POF specimen used. Generally, the polymer chains of the POF will be rearranged when the POF specimen is subjected to load. However, while the load is being removed, the unloading rate of 100 mm/min could be too fast for polymer chains to recover from their elongated structures to the original equilibrium conformations. This may result in retention of the retarded elastic deformations and the production of irrecoverable viscous flows which are due to polymer chains slipping past one another. The irrecoverable viscous flow dissipates the majority of its energy as heat, and exhibits the phenomenon known as hysteresis loops, as shown in Figure 4. When the next load-unload cycle begins, the elongated polymer chain will be stretched again, and the total retarded and irrecoverable permanent deformations increase with increasing load cycles.

**Figure 5.**
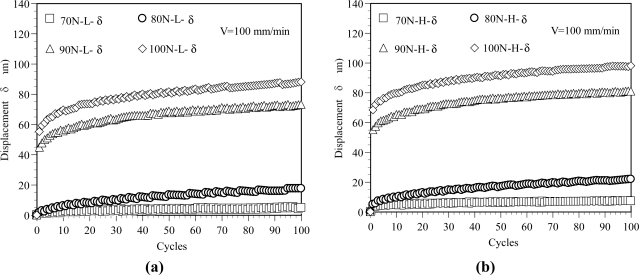
Variation of the displacement with the cycles at various load levels. (**a**) Displacement-cycles relations at L-Load. (**b**) Displacement-cycles relations at H-Load.

In the following discussions, the lowest load in the unloading process and the highest load in the elongation test process are denoted as L-Load and H-Load. The lowest load is 0 N in the elongation test process and the highest load in unloading process is defined as the load level in experiments. Figure 5 shows the variation of displacement with respect to the cycles for various load levels. The displacement-cycles curves obtained from the L-Load are shown in Figure 5(a) and the curves obtained from the H-Load are shown in Figure 5(b). The profile of the displacement curve for the L-Load is similar to the result for the H-Load. The results shown in Figure 5 indicate that the displacement increases with the increasing number of cycles. As the results in Figure 5(a) show, the total displacements can be obtained as 4.9, 17.6, 73.1 and 88.0 mm at 100 cycles for the L-Load with load levels of 70 N, 80 N, 90 N and 100 N, respectively. A higher load level results in a larger elongation in the POF specimen. The corresponding total displacements for the H-Load are 7.2, 21.9, 81.0 and 98.1 mm, as shown in Figure 5(b). Comparing the results shown in Figure 5(a,b), the H-Load has a larger displacement than L-Load does. This can be attributed to the retarded strain and viscous elastic strain of the polymer material.

**Figure 6.**
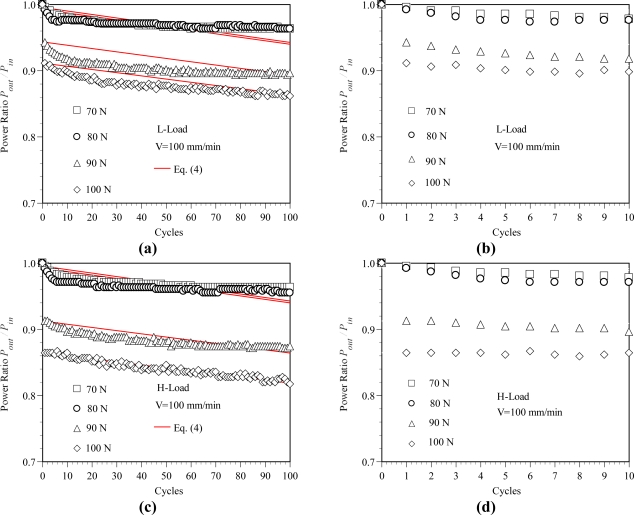
Variation of the power ratio with number of rollers. (**a**) Power ratio-cycles relations at L-Load. (**b**) An enlarged view for cycles 0–10. (**c**) Power ratio-cycles relations at H-Load. (**d**) An enlarged view for cycles 0–10.

The corresponding variations of the power ratio P_out_/P_in_ with the number of cycles are shown in Figure 6. As shown in the figure, the power ratio P_out_/P_in_ decreases with the increasing number of cycles or the increasing load level. The results shown in Figure 6(a) indicate that after 100 cyclic tests, the power ratio P_out_/P_out_ obtained from the L-Load decreases about 3.7, 4.1, 10.4 and 13.8% at the load levels of P = 70, 80 90 and 100 N, respectively. The corresponding power ratio P_out_/P_in_ obtained from the H-Load decreases about 3.7, 4.5, 12.6 and 18.3 %, respectively. It also can be found that the maximum power loss occurs at the first cyclic test, *i.e.*, C_V_ = 1. The results shown in Figure 6 indicate that the power losses are about 8.9% and 13.6%, respectively, for the L-Load and H-load at the load level P = 100 N and C_V_ = 1. It can be observed from the experimental cyclic load-displacement curves, as shown in Figure 4, that the maximum elongation δ occurs in the first cyclic test. Also, as shown in Figure 3, a larger elongation can result in a larger area under the load-displacement curve, *i.e.*, larger strain energy is accumulated in the deformed POF specimen. This leads to a larger power loss in the POF specimen [15]. In addition, it is observed that the displacement increases with the increasing number of cycles. However, the increase of displacement is slower and gradually reaches a stable value as the number of cyclic tests is greater than 60, as shown in Figure 6. Accordingly, the corresponding power ratio decreases, *i.e.*, power loss increases, with increasing cyclic tests. When the number of cyclic tests is greater than 60, the power losses of the POF specimens decrease very slightly.

Additionally, it is observed from experiments that the fiber profile becomes linearly tapered as the POF is elongated. The reduction of the core diameter near the necked portions of the fiber changes ray paths and therefore introduces a power loss as rays propagate through the linearly tapered regions. From the experimental results, it can be seen that the core diameter in the middle region of the POF specimen decreases as the elongation increases. In a previous study [16] of the effect of fiber elongation on the power loss in straight POFs, it was shown that the reduced core diameter in the linearly tapered region of the POF reduced the incident angle and therefore induced a greater power loss as a result of rays refracting outside the coating. Accordingly, as shown in Figure 5, a higher load level results in a larger elongation in the POF specimen and therefore induced a greater power loss, as shown in Figure 6. It is observed that the profiles of the power ratio *vs.* number of cycles for various load levels are similar and the maximum power ratio occurs at first cyclic test. Therefore, the power ratio obtained from the first cyclic test is denoted as η_o_ and used as a reference to normalize the ones obtained from the other tests. The normalized power ratio is defined as η̄ and can be expressed as:

(2) $$\bar{\eta }=(P_{\text{out}}/P_{\text{in}})/\eta_{o}$$

**Figure 7.**
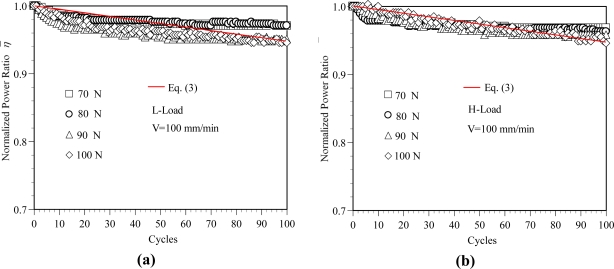
Variation of the normalized power ratio with the cycles at various load level. (**a**) Normalized power ratio-cycles relations at L-Load. (**b**) Normalized power ratio-cycles relations at H-Load.

The power ratios η_o_ for various load levels are listed in Table 1. The variation in the normalized power ratio η̄ with the number of cycles for various load levels is presented in Figure 7. It can be seen that the normalized power ratio curves are almost identical for various load levels. In other words, for a constant elongation rate, once the first cycle effect is removed, the normalized power ratio is essentially independent of the load level.

Applying a linear least squares fitting technique to the data plotted in Figure 7 for the SH-4001 POFs, the normalized power ratio η̄ can be related to the number of cycles via the following expression:

(3) $$\bar{\eta }=1-0.00053C_{y}$$

The power ratio related to the number of cycles can be expressed as the following expression:

(4) $$\eta =\eta_{o}\times (1-0.00053C_{y})$$

The predicted results obtained using Equation (4) for the variations of the normalized power ratios with respect to the cycles for various load levels are shown in Figure 6 (indicated by the continuous lines). Comparing the two sets of results in each figure, it is found that the difference between them is no more than 3% in each case. The proposed empirical expression can thus provide a good prediction of the power loss as the POF is subjected to cyclic tensile loading.

## 5. Conclusions

This study has investigated the effects of the cyclic load level and number of cycles on the power loss in polymer optical fibers deformed at load levels between 70 and 100 N. The results show that the POF specimen is significantly affected by the number of cycles and the load level. The power ratio reduces significantly as the number of cycles or the load level increases. The power loss can reach as high as 18.3% after 100 cyclic loadings. Based on the experimental results, an empirical expression is formulated to relate the power loss with the number of cycles. The maximum deviation between the predicted power loss obtained from the proposed equation and the experimental result is found to be less than 3%. Thus, the suitability of the relative displacement as a means of predicting the power loss in deformed POF sensors is confirmed. The potential applications of the developed cyclic tensile-POF sensing element can be found in measuring small displacements in certain areas or landslide alarming for its high sensitivity under cyclic loading deformation.

## Figures and Tables

**Table 1.** The power ratio η_o_ at C_y_ = 1 for various load levels.

	**L-Load**	**H-Load**
70 N	0.995	0.995
80 N	0.992	0.992
90 N	0.943	0.912
100 N	0.911	0.864
